# Tunable Low Energy, Compact and High Performance Neuromorphic Circuit for Spike-Based Synaptic Plasticity

**DOI:** 10.1371/journal.pone.0088326

**Published:** 2014-02-13

**Authors:** Mostafa Rahimi Azghadi, Nicolangelo Iannella, Said Al-Sarawi, Derek Abbott

**Affiliations:** School of Electrical and Electronic Engineering, The University of Adelaide, Adelaide, South Australia, Australia; The University of Plymouth, United Kingdom

## Abstract

Cortical circuits in the brain have long been recognised for their information processing capabilities and have been studied both experimentally and theoretically via spiking neural networks. Neuromorphic engineers are primarily concerned with translating the computational capabilities of biological cortical circuits, using the Spiking Neural Network (SNN) paradigm, into *in silico* applications that can mimic the behaviour and capabilities of real biological circuits/systems. These capabilities include low power consumption, compactness, and relevant dynamics. In this paper, we propose a new accelerated-time circuit that has several advantages over its previous neuromorphic counterparts in terms of compactness, power consumption, and capability to mimic the outcomes of biological experiments. The presented circuit simulation results demonstrate that, in comparing the new circuit to previous published synaptic plasticity circuits, reduced silicon area and lower energy consumption for processing each spike is achieved. In addition, it can be tuned in order to closely mimic the outcomes of various spike timing- and rate-based synaptic plasticity experiments. The proposed circuit is also investigated and compared to other designs in terms of tolerance to mismatch and process variation. Monte Carlo simulation results show that the proposed design is much more stable than its previous counterparts in terms of vulnerability to transistor mismatch, which is a significant challenge in analog neuromorphic design. All these features make the proposed design an ideal circuit for use in large scale SNNs, which aim at implementing neuromorphic systems with an inherent capability that can adapt to a continuously changing environment, thus leading to systems with significant learning and computational abilities.

## Introduction

Brain processes large amounts of data in real-time in the presence of noise, while consuming little power. The brain also takes little space and has extraordinary processing features. The ultimate goal for neuromorphic engineers is to develop a cybernetic system, which closely mimics the capabilities of the brain. To reach this goal, understanding and implementing *in silico* the main components of cortical networks, i.e. neurons and synapses, is a crucial first step.

Currently, the dynamical behaviour of biological neurons is best understood through biophysically detailed models, such as the Hodgkin-Huxley (HH) model [1], which given the correct parameters, can replicate various experimentally observed response properties. Using such models one can develop hypotheses about cortical circuit behaviour and any underlying computations taking place. The complexity of such biophysical models can be a prohibitive bottleneck when translation into silicon is desired. For this reason simpler models, such as the Integrate-and-Fire (IF) [2], [3], have been adopted in simulating networks, even though they lack the dynamic realism of real cortical circuits.

In addition to neurons, synapses are the second main building blocks of SNNs. Similar to neurons, synapses also have complex structures and behaviours. They are widely thought to be the essential components responsible for learning and memory in neural networks [4]. Synapses alter their strength or efficacy through activity-dependent biophysically driven changes coordinated by pre-synaptic activities or by both pre- and post-synaptic activities. To date, the precise molecular mechanisms underlying how synapses change their efficacy requires further elucidation. However, there exists a significant number of hypotheses that aim to approximate synaptic efficacy alterations [5]. These hypotheses that govern the synaptic weight changes, are so called synaptic plasticity rules. Generally, these rules can be divided into two main groups, namely short-term and long-term plasticity. While long-term plasticity is believed to be the underlying mechanism for learning and memory, short-term plasticity is responsible for decoding and processing neural signals on short-time scales [6]. The short-term plasticity mechanisms including excitatory and inhibitory depression and facilitation has been successfully implemented and observed in VLSI technology [7], [8]. The focus of this paper is on STDP, which is a long-term synaptic plasticity rule.

Identical to neuron models, there are a variety of synaptic plasticity models. Some of these models embrace certain features of real biological synapses, however they tend to be complex in their (mathematical) formulation. On the other hand, other models have been mathematically formulated to replicate the outcomes of a subset of known experiments. Their representation is typically simpler in form allowing, in some cases, reduced problematic translation into silicon. Generally, the main purpose of such simplified rules is to mimic, as accurately as possible, the outcomes of various experimental synaptic plasticity protocols.

In this paper, we propose a new Very Large Scale Integration (VLSI) implementation of a malleable synaptic circuit that is capable of mimicking the outcomes of various synaptic plasticity experiments. We demonstrate that the new design has a compact structure and possesses low power consumption, which is required for VLSI implementations of large-scale spiking neural networks. In addition, the robustness of the proposed circuit is verified against transistor mismatch and process variations. The results show that the new circuit is a fairly stable design in terms of transistor mismatch. These features make this new design an ideal learning component that may benefit various VLSI synaptic plasticity systems. The proposed circuit is of potential interest for future large scale neuromorphic circuits with significantly high numbers of neurons and synapses, where low power consumption, compactness, accuracy and mismatch tolerance are absolutely essential.

## Materials and Methods

Various plasticity rules have been proposed throughout the literature. In order to achieve a fair comparison among these rules, we compare them from two aspects. Firstly, their capability in reproducing various synaptic plasticity experiments, and secondly their simplicity and suitability to be employed in large-scale neural simulations, and/or large-scale hardware realisations. Here, a variety of experimental protocols are briefly summarized, in order to provide the reader with an understanding of various conditions under which synaptic plasticity rules are simulated and compared. In the following sections, first some important synaptic plasticity protocols are reviewed and their structures are described. And second, some significant synaptic plasticity models are reviewed and their structures and various synaptic plasticity abilities are highlighted. Then, we introduce our new proposed circuit, which is based on one of the reviewed synaptic plasticity rules.

### Synaptic Plasticity Experiments

To study both the outcome and underlying cause of plastic changes in synapses, experimentalists have resorted to carefully crafted hypotheses and stimulation paradigms to test and characterize physiological changes of synapses. Understanding these alterations with respect to activities of the pre- and post-synaptic neurons and their corresponding dynamics have shed light on how neural activity affect synaptic strength and bring about Long Term Potentiation (LTP) or Long Term Depression (LTD) [9]. This permits neuroscientists to describe the behaviour of the synapse with a mathematical expression, and assists them in developing a detailed model for synaptic plasticity.

In order to measure, the efficiency of a model or a circuit in replicating the outcomes of experiments, one can define an error function based on the difference between the weight changes predicted by a candidate model or circuit, and those of the biological experiments. An instance of such a measure, is the Normalized Mean Square Error (NMSE) function proposed and utilised in [10]. The NMSE is calculated using the following equation:
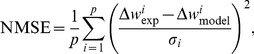
(1)where 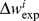, 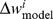 and 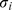 are the mean weight change obtained from biological experiments, the weight change obtained from the model or circuit under consideration, and the standard error mean of 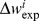 for a given data point 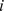, respectively; 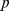 represents the number of data points in a data set under consideration. In order to minimize the resulting NMSEs for the model/circuit and fit their output to the experimental data, there is a need to adjust the model or circuit bias parameters and time constants. This is an optimisation process of the model parameters/circuit biases, which results in reaching a minimum NMSE value and so a close fit to the experimental data.

With respect to this error measure, an ideal synaptic plasticity model/circuit is therefore the one that can reproduce the outcomes of a large number of biological experiments, while examined, and while achieving the smallest possible error. Hence, the replication of plasticity outcomes a single model can account for is a desirable measure/benchmark on model performance. In the following, we review some of these experimental protocols, which have been utilised in this paper, to verify the functionality and performance of the proposed circuit.

#### Pairing protocol

The pair-based Spike Timing Dependent Plasticity (STDP) protocol has been extensively used in electrophysiological experiments and simulation studies [11], [12]. In this protocol, 60 pairs of pre- and post-synaptic spikes with a delay of 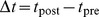 are conducted with a repetition frequency of 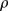 Hz (in many experiments 1 Hz repetition frequency is used). This experimental protocol has been utilised in experiments reported in [11], [13], [14], and also has been employed in simulations and circuit designs for synaptic plasticity [15]–[17].

#### Frequency-dependent pairing protocol

In the simple pairing protocol, the repetition frequency of spike pairs is kept constant. However, it has been illustrated in [18] that altering the pairing repetition frequency affects the total change in weight of the synapse. It is shown that in higher pairing frequencies, the order of pre-post or post-pre spike pairs does not matter and both cases will lead to LTP. However, in lower pairing frequencies, pre-post results in LTP and post-pre combination results in LTD [4], [18].

#### Triplet protocol

There are two types of triplet patterns that are used in the hippocampal experiments, which are also adopted in this paper to compute the prediction error as described in [10]. Both of them consist of 60 triplets of spikes, which are repeated at a given frequency of 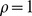 Hz. The first triplet pattern is composed of two pre-synaptic spikes and one post-synaptic spike in a pre-post-pre configuration. As a result, there are two delays between the first pre and the middle post, 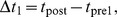 and between the second pre and the middle post 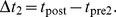 The second triplet pattern is analogous to the first but with two post-synaptic spikes, one before and the other one after a pre-synaptic spike (post-pre-post). Here, timing differences are defined as 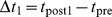 and 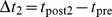.

#### Extra triplet protocol

In addition to the aforementioned triplet protocol employed in [10], which considers only two combinations of spike triplets, there are other combinations (rather than pre-post-pre or post-pre-post) of spikes triplet which have not been explored in [10], but have been used in another set of multi-spike interaction experiments performed in [13]. The experimental triplet protocol as described in [13] is as follows; a third spike is added either pre- or post-synaptically to the pre-post spike pairs, to form a triplet. Then this triplet is repeated 60 times at 0.2 Hz to induce synaptic weight changes. In this protocol, there are two timing differences shown as 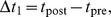 which is the timing difference between the two most left pre-post or post-pre spike pairs, and 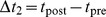, which is the timing difference between the two most right pre-post or post-pre spike pairs.

#### Quadruplet protocol

This protocol is composed of 60 quadruplets of spikes repeated at frequency of 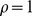 Hz. The quadruplet is composed of either a post-pre pair with a delay of 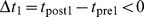 precedes a pre-post pair with a delay of 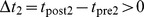 with a time 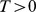, or a pre-post pair with a delay of 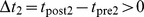 precedes a post-pre pair with a delay of 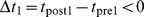 with a time 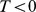, where 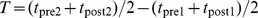. In other words, in the case of 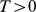, post1-pre1 spike pair precedes pre2-post2 pair. However, in the case of 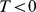, pre2-post2 precedes post1-pre1, in the experimental protocol definition [10]. Identical to [10], in all quadruplet experiments in this paper, 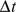 = 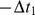 = 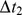 = 5 ms.

#### Poissonian protocol for the BCM rate-based learning

In order to test the ability of the targeted timing-based plasticity rules and the proposed spike timing-based synaptic plasticity circuit in generating spike rate-based learning rule which mimics the effects of Bienenstock-Cooper-Munro (BCM) rule, a Poissonian rate-based experimental protocol is also employed. Under this protocol, the pre-synaptic and post-synaptic spike trains are generated as Poissonian spike trains with firing rate of 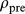 and 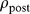, respectively. This is the same protocol that has been used in [10] to show how their proposed TSTDP model can present a close mapping to the BCM model. This paper utilizes a similar protocol to stimulate the proposed TSTDP circuit and examines if it is capable of reproducing a similar BCM-like behaviour.

### Synaptic Plasticity Rules

Although there are a variety of synaptic plasticity rules and experiments, here we only review STDP rules, which are used in the implementation of the proposed neuromorphic VLSI circuit.

#### Pair-based STDP

The pair-based STDP rule is the most popular form of STDP that has been investigated in many computational studies e.g. [12], [19], [20]. In addition, it has been also widely used in VLSI implementations [15], [16], [21]–[25]. This rule is represented as
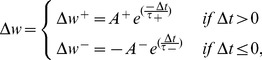
(2)where 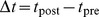 is the timing difference between a single pair of pre- and post-synaptic spikes. As shown in Eq. 2, if 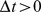, namely if a pre-synaptic spike precedes a post-synaptic one in a specified time window (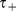), an increase in the synaptic weight takes place. On the other hand, if a pre-synaptic spike arrives in a determined time window (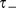) after a post-synaptic one (i.e. 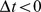), it leads to a decrease in the synaptic weight. The magnitude of these increase and decrease is determined as a function of 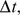 as well as potentiation and depression amplitude constants (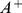 and 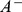, respectively) [19].

#### Triplet-based STDP

The weight changes in this model of synaptic plasticity occur according to the timing differences among triplet of spikes in contrary to the pair-based STDP, which alters the synaptic weight based on the timing differences between pairs of spikes. The triplet-based STDP (TSTDP) rule is described by
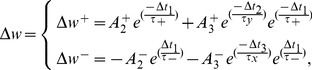
(3)where the synaptic weight can be decreased (depressed) if a pre-synaptic spike occurs, or can be increased (potentiated) at the time when a post-synaptic spike arrives. Here, 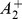, 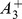 and 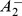, 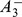 are the potentiation and depression amplitude parameters, respectively. In addition, 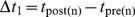, 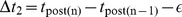 and 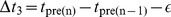, are the time differences between combinations of pre- and post-synaptic spikes, while 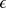 is a small positive constant, which ensures that the weight update uses the correct values occurring just before the pre or post-synaptic spike of interest. In Eq. 3, 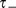 and 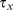 are depression time constants, while 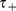 and 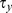 are potentiation time constants [10].

Since the TSTDP rule utilises higher order temporal patterns of spikes, it is shown to be able to account for the outcomes of several experimental protocols including the frequency-dependent pairing experiments performed in the visual cortex [18], or triplet, and quadruplet spike experiments performed in the hippocampal [14]. Note that, the PSTDP rule fails to reproduce the outcomes of these experiments. This is due to a linear summation of the effect of potentiation and depression in the PSTDP rule, while the underlying potentiation and depression contributions in the TSTDP rule, do not sum linearly [13].

Numerical simulation results presented in [10] demonstrate how a minimized version of the full TSTDP rule, which is shown in Eq. 3, can approximate a number of biological experiments performed in hippocampal including quadruplet, triplet and STDP window experiments outcomes. This minimised TSTDP rule is presented as
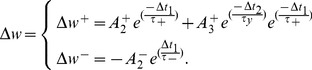
(4)


This model is able to account for quadruplet, triplet, and pairing (window) experiments as shown in [10], [17]. In addition to the capability of simultaneously approximation of triplet, quadruplet and STDP window experiments with the same set of synaptic parameters, another minimal version of TSTDP rule, is also capable of reproducing the results of the frequency-dependent pairing experiments performed in the visual cortex [18]. The minimal model for this experiment can be shown as
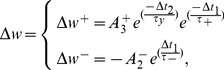
(5)which is simpler and utilizes a lower number of synaptic parameters, and therefore needs a new set of parameters, in comparison with the previous minimal model for hippocampal experiments.

Besides the ability of reproducing timing-based experiments, the TSTDP rule has the capability to demonstrate BCM-like behaviour. The BCM learning rule is an experimentally verified [26], [27] spike rate-based synaptic plasticity rule, proposed in 1982 [28]. Unlike STDP, which is a spike-timing based learning rule, synaptic modifications resulting from the BCM rule depends on the rate (activity) of the pre- and post-synaptic spikes [28].

This paper proposes a novel VLSI design for TSTDP rule, with a fewer number of transistors, smaller area, and lower power consumption, than all previously published circuits, yet with all their synaptic capabilities. These features make this design an ideal learning component for large scale neuromorphic circuits. We will show that the proposed circuit is able to faithfully reproduce the outcomes of many biological experiments, when examined under experimental protocols mentioned earlier.

### Proposed VLSI Implementation for the TSTDP Rule

The proposed design is implemented based on a different arrangement of the TSTDP rule presented in Eq. 3. This new arrangement is given by
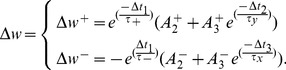
(6)


**Figure 1:**
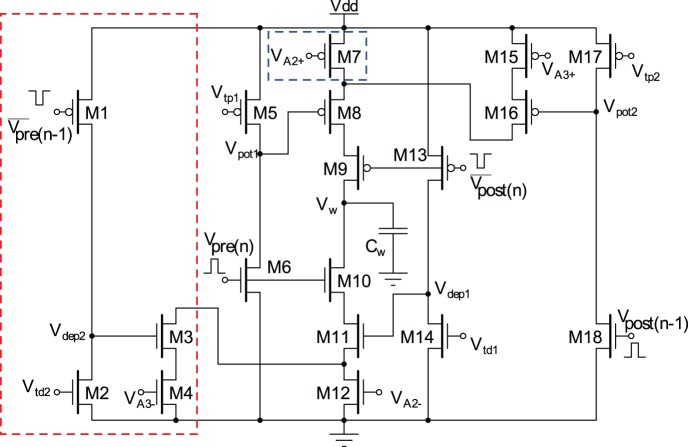
Proposed circuit for the full TSTDP rule shown in Eq. 6. The circuit for the first minimal TSTDP model does not include transistors M1–M4 shown in the red dashed-box. Furthermore, the second minimal TSTDP circuit, does not include the M1–M4 transistors, nor the M7 transistor, shown in the blue dashed-box. Therefore, the source of M8 will be connected only to the drain of M16, in both minimal circuits.



The new TSTDP circuit is demonstrated in Fig. 1. This symmetric circuit operates as follows: When a pre-synaptic spike, 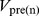, is received at the gate of M6 at 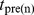, 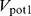 reaches ground resulting in switching on M8, and then starts to increase linearly toward 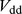. The rate of this increase is determined by 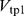 that is applied to the gate of M5, and corresponds to the pairing potentiation time constants, 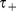, which is present in both pairing and triplet potentiation terms as shown in the first line of Eq. 3. In fact, 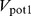 is a triangular voltage, which is controlled by the leaky integrator composed of the output conductance of M5 and the gate capacitor of M8, to control the existence of the potentiation in the first place and allows a current, 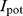, to flow through the potentiation branches (M7–M9 and/or M15–M16-M8–M9) at the time of arrival of a post-synaptic spike at M9, 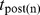. The linear increase of 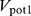, which starts at 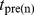, and leads to charging the weight capacitor through M8 once 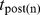 arrives, is approximately proportional to

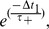
where 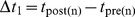 and 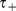 approximates by 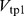. This term is repeated twice in the first line of Eq. 3, and can be factorised as it is shown in the first line of Eq. 6.

Furthermore, the addition term shown in the second term of first line of Eq. 6 that determines the amount of potentiation as a result of both pair and triplet interactions, is approximated through a sum of two currents that charge the weight capacitor, 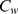, and represent synaptic weight potentiation. The first current is controlled by the controllable voltage 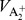, while the second one is determined by both the second potentiation dynamic 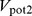, as well as the controllable voltage 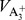. This voltage depends on the arrival time of the previous post-synaptic spike, 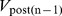. When a post-synaptic spike arrives at M18, 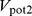 reaches ground and after the post-synaptic pulse duration is finished, it starts to increase linearly toward 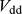. The rate of this increase is determined by 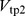 that is applied to the gate of M17, and corresponds to the triplet potentiation time constants, 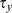. Therefore, the current flowing through M15–M16 can be an approximation of
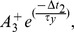
where 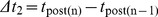. The current flowing through M15–M16 transistors accumulates with the current flowing through M7 transistor (which is controlled by gate voltage 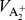) and forms the total current that is approximately proportional to



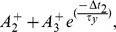
and it represents an approximation of the second term of the first line of Eq. 6.

The same dynamic operates in the depression half of the proposed circuit, in which currents flow away from the weight capacitor, 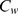, and represents synaptic weight depression. In this part, current sinks away from the weight capacitor through M10–M12, if there has been a pre-synaptic action potential that arrives at M10, in a specified time window defined by 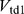 (which corresponds to 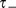), after a post-synaptic spike arrives at M13. The amount of this current is first determined by the time difference between the pre- and post-synaptic spikes (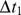) and then by the controllable voltage, 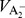. Therefore, this current approximates
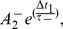
where 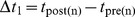. This is the pairing depression current that flows away from the weight capacitor and results in depression due to post-pre spike pairs.

In addition, another current that can discharge the capacitor and results in depression, will flow through M10–M11–M3–M4, if two conditions are satisfied. First, if there has been a previous pre-synaptic spike, 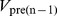, in a specified time window, set by 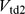 (which corresponds to 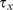), before the current pre-synaptic spike, 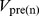, arrives at M10 gate. And second, if a post-synaptic spike arrived at M13 gate in a specified time window set by 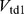 before the current and after the previous pre-synaptic spikes. The magnitude of this current is first controlled by the time difference between the pre- and post-synaptic spikes (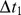), second with the time difference between the 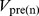 and 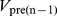 spikes, (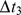), and then by controllable voltage, 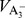. Therefore, this current approximates
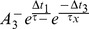
where 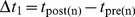 and 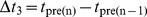. This is the triplet depression current that flows away from the weight capacitor and results in depression due to pre-post-pre spike triplet.

If the above two currents accumulate together, they form the depression term of both Equations 3 and 6 which are equal as follows
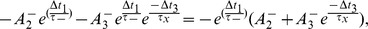
where the negative sign represents that the current is depressive and shows that it flows away from the weight capacitor.

Note that the above explanations contain assumptions that approximate the TSTDP rule using our proposed circuit. However, from a circuit analysis point of view, if M3–M4, M7–M12, and M15–M16 operate in the subthreshold regime [29], the analytical expressions for 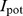 and 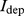, which are potentiation and depression currents, respectively are as follows
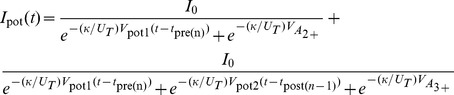
(7)




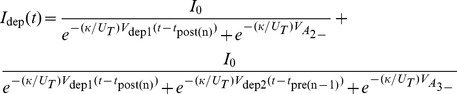
(8)where 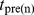 and 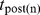 are current pre- and post-synaptic spike times respectively, while 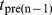 and 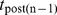 are the times at which the previous pre- and post-synaptic spikes have arrived. Therefore, the voltage change in synaptic weight, shown as 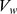 in Fig. 1, is approximated as:
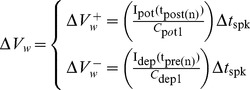
(9)where 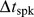 are the width of pre- and post-synaptic spike pulses, and 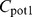 and 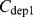 are the parasitic capacitance available at the gate of M8, and M11, respectively. Please note that, as in the proposed circuit, similar to the TSTDP model, whenever a pre-synaptic spike arrives at 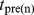, a depression can happen, while a potentiation can happen whenever a post-synaptic spike arrives. This analysis is similar to the analytical method utilised in [16].

Below, experimental results of the proposed circuit are presented and compared with previous synaptic plasticity circuits. Furthermore, the circuit is also compared with other synaptic plasticity circuits in terms of power consumption, area and ability in reproducing the outcomes of various biological experiments.

## Results and Discussion

### Experimental Setup

This section provides information about the experimental setup, under which simulations are performed. These simulations are carried out, in order to verify the performance of the proposed circuit and compare it with published synaptic plasticity circuits in the literature.

#### Minimal TSTDP circuits

As already discussed, in order to regenerate the outcomes of several biological experiments, minimal models of the TSTDP rule, shown in Eqs. 4 and 5 are sufficient. Matlab simulation results of the first minimal model, presented in [10] demonstrate that the first minimal TSTDP model, shown in Eq. 4, can efficiently generate STDP window, triplet, and quadruplet experiments, using the synaptic parameters optimised for these experiments. In addition, according to another set of numerical simulations, the frequency-dependent pairing experiments and also the BCM-like rate-based experiments, can be regenerated through the second minimal model, shown as Eq. 5, and by employing the synaptic parameters optimised for the frequency-dependent pairing experiments. As the full TSTDP rule is minimised, the proposed circuit that approximates the full TSTDP rule, can also be further modified and hence the number of transistors is reduced from the 18 transistors required for the full TSTDP circuit shown in Fig. 1.

This paper presents experimental results of two minimal TSTDP circuits that correspond to the two aforementioned minimal TSTDP models presented in [10]. According to the minimal rules shown in both Eqs. 4 and 5, the depression contribution of the spikes triplet interactions can be neglected without having a significant effect on the circuit performance in reproducing the targeted biological experiments. The triplet depression part in the full TSTDP circuit shown in Fig. 1, is the four transistors surrounded in the red-dashed box. Therefore, the minimal TSTDP circuit, is the one shown in Fig. 1 minus the part enclosed in the red-dashed box, i.e only 14 transistors are needed to regenerate all desired biological experiments. This is the first minimal TSTDP circuit.

In addition, the numerical simulation results suggest that, for generating the frequency-dependent pairing experiments, as well as the BCM experiment, the pairing potentiation part is not necessary and can be removed. Therefore, in the case of second minimal TSTDP rule, shown in Eq. 5, 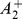 can be zeroed. As a result, one more transistor that is shown in the blue dashed-box can be also removed from the proposed circuit and therefore only 13 transistors are required for generating the mentioned pairing and BCM experiments [10]. This is the second minimal TSTDP circuit.

#### Experiments data sets

Since there are two versions of the minimal TSTDP rule, two sets of simulations have been performed using the proposed minimal circuits. Each simulation set considers a specific set of data from the experiments. The first experimental data set that was utilized originates from hippocampal culture experiments that examine pairing, triplet and quadruplet protocols effects on synaptic weight change [14]. This first data set consists of 13 data points obtained from Table 2 of [10]. These data points include (i) two data points and error bars for pairing protocol (ii) three data points and error bars for quadruplet protocol, and (iii) eight data points and error bars for triplet protocol. This data set shows the experimental weight changes, 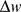 *s*, as a function of the relative spike timing 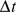, 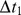, 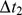 and 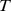 under pairing, triplet and quadruplet protocols in hippocampal culture. The second data set originates from experiments on the visual cortex, which investigated how altering the repetition frequency of spike pairings affects the overall synaptic weight change [4], [18]. This data set is composed of 10 data points (obtained from Table 1 of [10]) that represents experimental weight change, 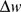, for two different 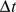
’s, and as a function of the frequency of spike pairs under a frequency-dependent pairing protocol in the visual cortex. The data set is composed of those 10 black data points and error bars that were used in numerical simulations using the TSTDP minimal model reported in [10].

**Table 1:** Optimised biases for the minimal TSTDP circuits and two data sets.

Data set	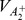 (V)	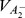 (V)	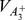 (V)	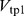 (V)	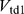 (V)	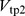 (V)	NMSE
Hippocampal (first)	3.2	0.32	2.7	2.75	0.35	2.65	2.04
Visual cortex (second)	0	0.29	2.7	2.7	0.17	2.86	0.39

Values of bias parameters for the minimal circuit that have been optimised in order to reach the minimal NMSEs for the targeted set of data and experiments. Hippocampal (first) set of bias parameters generate the results shown for pairing, quadruplet and triplet experiments. Visual cortex (second) set of bias parameters are optimised for reaching the minimal NMSE in frequency-dependent pairing experiment, as well as rate-based BCM experiments.

**Table 2:** Comparison of various synaptic plasticity VLSI circuits.

Plasticity Circuit\Experiment	STDP window	Pairing frequency	Triplet	Quadruplet	BCM
PSTDP [15]	**√**	**×**	**×**	**×**	**√**
PSTDP [21]	**√**	**×**	**×**	**×**	**√**
PSTDP [16]	**√**	**×**	**×**	**×**	**√**
PSTDP [30]	**√**	**×**	**×**	**×**	**√**
PSTDP [51]	**√**	**×**	**×**	**×**	**√**
PSTDP [47]	**√**	**×**	**×**	**×**	**√**
PSTDP [22]	**√**	**×**	**×**	**×**	**√**
PSTDP [49]	**√**	**×**	**×**	**×**	**√**
PSTDP [52]	**√**	**×**	**×**	**×**	**√**
PSTDP [25]	**√**	**×**	**×**	**×**	**√**
PSTDP [39]	**√**	**×**	**×**	**×**	**√**
SDSP [36]	√*	√**	√**	√**	√**
Voltage-based BCM [23]	**√**	**√**	**√**	√*	**√**
Iono-neuromorphic [24]	√*	√**	√**	√**	√**
Iono-neuromorphic [37]	√*	√**	√**	√**	**√**
TSTDP [32]	**√**	**√**	**√**	**√**	**√**
TSTDP [38]	**√**	**√**	**√**	**√**	**√**
TSTDP [17]	**√**	**√**	**√**	**√**	**√**
Proposed TSTDP	**√**	**√**	**√**	**√**	**√**

**√** means that the outcomes of experiments can be closely mimicked using the circuit.

√*means that the related study has not investigated the corresponding experiment, but according to its plasticity rule, it can most likely reproduce the expected experiment, though using a different set of plasticity parameters.

√**means that the related study has not investigated the corresponding experiment, but according to its plasticity rule, it might be able to reproduce the expected experiment.

**×**means that the outcomes of experiments cannot be generated using the circuit.

#### Circuit simulation and configuration

The minimised circuits are simulated in HSpice using the 0.35 µm C35 CMOS process by AMS. All transistors in the design (shown in Fig. 1) are set to 1.05 µm wide and 0.7 µm long. The weight capacitor value is set to 1 pF. It should be noted that the circuits are simulated in an accelerated time scale of 1000 times compared to real time, with all pulses having a 1 µs pulse width. This is the same approach that has been utilised by previous synaptic plasticity circuit implementations such as [8], [22], [23], [30], [31]. For the sake of simplicity when comparing simulation results to the biological experimental data, all shown results are scaled back to real time. Furthermore, the nearest-spike interaction of spikes is implemented in the proposed circuit that corresponds to the nearest-spike model of TSTDP rule presented in [10]. The circuit is examined under same protocols, using which the biological experiments and the Matlab numerical simulations were carried out.

#### Data fitting approach

Identical to [35], and previous TSTDP circuit studies [17], [32], which test their proposed triplet model/circuit simulation results against the experimental data using a Normalized Mean Square Error (NMSE) for each of the data sets, the proposed circuit is verified by comparing its simulation results with the experimental data and ensuring a small NMSE value. The NMSE is calculated using Eq. 1. In order to minimize the resulting NMSEs for the circuit and fit the circuit output to the experimental data, there is a need to adjust the circuit bias parameters and time constants. This is an optimisation process of the circuit bias voltages, which results in reaching a minimum NMSE value and so the closest possible fit to the experimental data.

#### Circuit bias optimisation method

In order to minimise the NMSE function and achieve the highest analogy to the experimental data, the circuit bias voltages, which tunes the required parameters from the models should be optimised. For this purpose, Matlab and HSpice were integrated in a way to minimise the NMSE resulted from circuit simulations using the Matlab built-in function fminsearch. This function finds the minimum of an unconstrained multi-variable function using a derivative-free simplex search method.

### Synaptic Plasticity Experiments with the Proposed TSTDP Minimal Circuits

#### Pairing experiment (STDP timing window)

**Figure 2:**
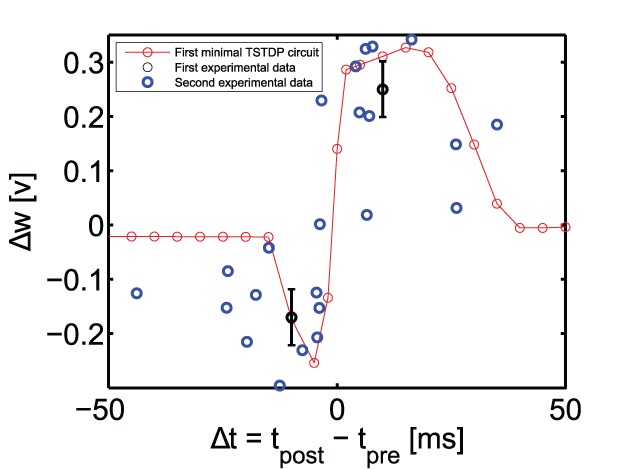
STDP timing window experiment in the hippocampal region can be approximated using the minimal TSTDP circuit. Simulation results are produced under pairing protocol and using the first minimal TSTDP circuit. The circuit bias parameters for generating the window approximation are those corresponding to the hippocampal data set shown in Table 1. The first experimental data set shown in black contains two data points with their standard error mean extracted from [10], and the second experimental data set is part of the normalised experimental data extracted from [11].

The first simulation that is performed using the proposed minimal TSTDP circuit, which does not include M1–M4 shown in Fig. 1, is reproducing the STDP learning window that demonstrates spike timing dependent potentiation and depression, under pairing protocol. Fig. 2 shows how the proposed circuit can successfully perform the timing dependent weight modifications. This figure shows the normalised experimental data extracted from [11] in blue. It suggests that the proposed circuit behaviour under a pairing (window) protocol can approximate the experimental data generated with the same protocol. Beside the blue experimental data, two other experimental values for 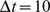 and 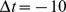 are shown with their standard error mean represented by black bars. These points are the first two points of the 13 data points of the aforementioned first (hippocampal) data set. These two points, were utilised to test and optimise the bias voltages of the first minimal TSTDP circuit. This is a similar approach to the method used in [10].

#### Quadruplet experiment

**Figure 3:**
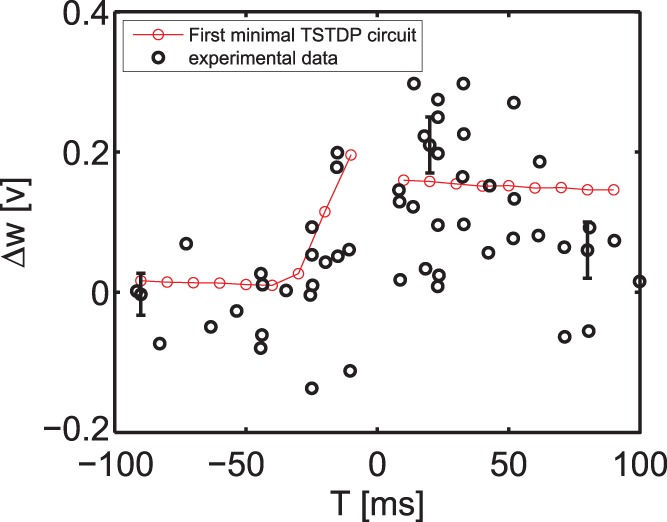
Quadruplet experiment in the hippocampal region can be approximated using the minimal TSTDP circuit. Simulation results are produced under quadruplet protocol and using the first minimal TSTDP circuit. The circuit bias parameters for generating the quadruplet approximation are those corresponding to the hippocampal data set as shown in Table 1. The experimental data shown in black were extracted from [14].

The second simulation is performed using the first minimal TSTDP circuit and under quadruplet protocol. Fig. 3 demonstrates how the proposed circuit approximates the timing dependent weight modifications close to those for quadruplet experiment. In these results, the black data points are extracted from [14], and the black deviation bars and data points are those that were used in [10] for quadruplet experiments. The circuit bias parameters for generating the quadruplet approximation are those corresponding to the hippocampal data set shown in Table 1.

#### Triplet experiment

**Figure 4:**
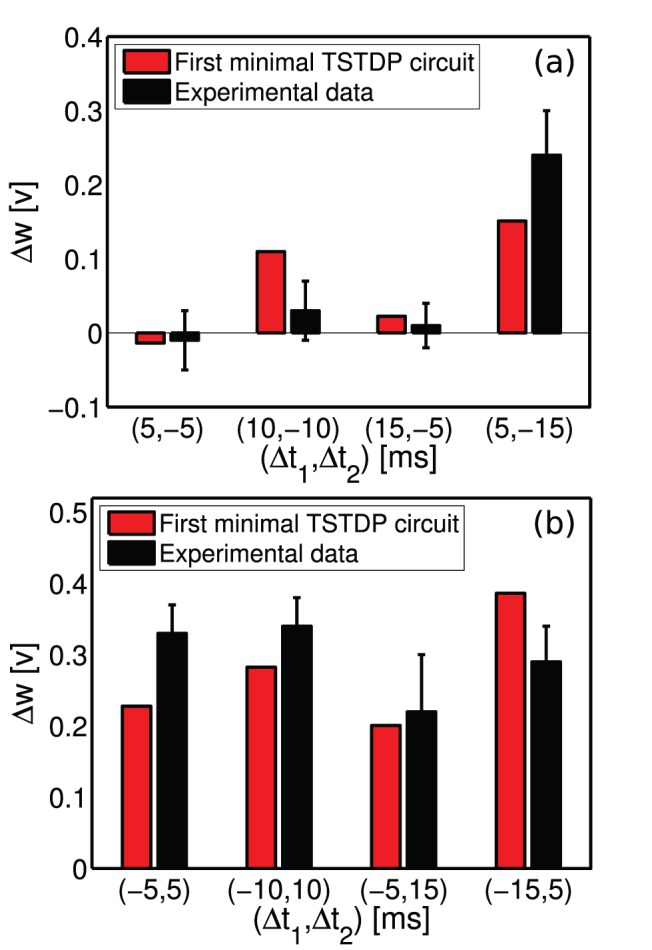
Triplet experiments in the hippocampal region can be approximated using the minimal TSTDP circuit. Simulation results are produced under the triplet protocol, and using the first minimal TSTDP circuit. The circuit bias parameters for generating the triplet approximation are those corresponding to the hippocampal data set as shown in Table 1. The experimental data, shown in black and their standard deviations extracted from [10], [14]. (a) Simulation and experimental results for the pre-post-pre combination of spike triplets with various timings. (b) Simulation and experimental results for the post-pre-post combination of spike triplets with various timings.

The third experiment that is performed on the first minimal TSTDP circuit is the triplet experiment performed in the hippocampal region and reported in [10], [14]. Fig. 4 demonstrates how the proposed circuit approximates the timing dependent weight modifications close to those for triplet experiments. In the shown results, the black data and deviation bars are those that were used in [10], [14] for triplet experiments. The circuit bias parameters for generating the triplet approximation are those corresponding to the hippocampal data set as shown in Table 1.

Simulation results show that the TSTDP circuit can distinguish between the pre-post-pre and post-pre-post spike combinations and show analogy to the experiments. However, the simulation results using the computational PSTDP model shown in [10], as well as the results generated using a PSTDP circuit [32], demonstrate that the pair-based STDP models and circuits do not have the ability to distinguish among triplet combinations.

Simulation results of the minimal TSTDP circuit using the first set of data, hippocampal experimental data [14], suggest that the proposed minimal circuit, can reach a good approximation of pairing, quadruplet, and triplet experiments, using a shared optimised set of bias voltages. Using these bias voltages a 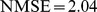 is obtained, when considering the 13 data points in the hippocampal data set. This is better than the minimal NMSE obtained using the minimal TSTDP computational model, as presented in [10].

In addition to the above experiments that are similar to the experiments performed by Pfister and Gerstner in [10], the proposed minimal circuit is additionally tested for all possible combination of spike triplets under the same protocol that used by Froemke and Dan [13], [33].

#### Extra triplet experiment

**Figure 5:**
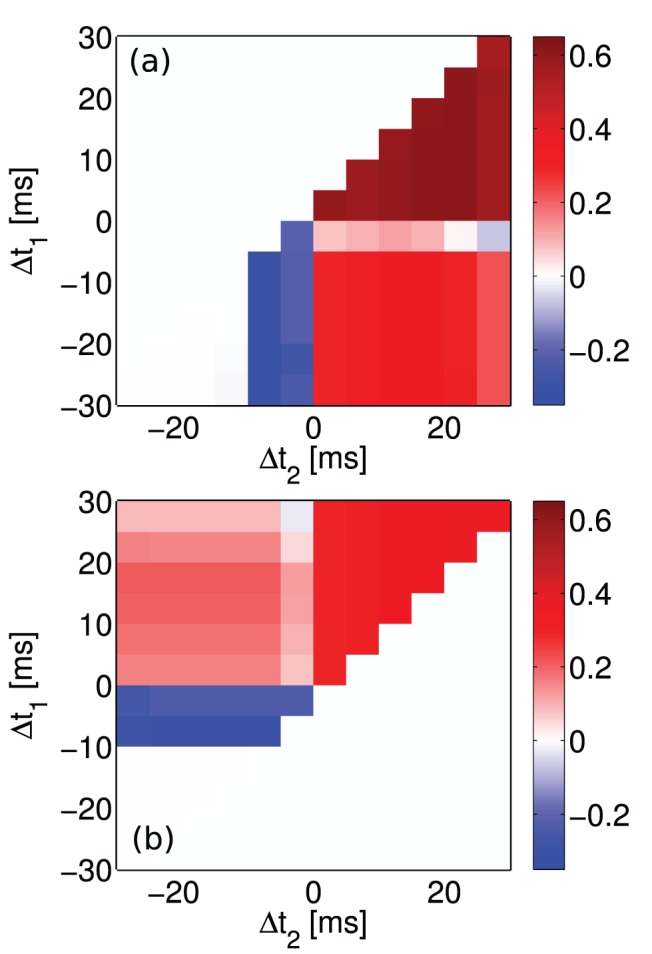
Extra triplet experiments using the suppression STDP model performed in [13] can be approximated using the minimal TSTDP circuit. Synaptic weight changes in result of extra triplet protocol for (a) pre-post-post (top right triangle), post-post-pre (bottom left triangle) and post-pre-post (right bottom square) and (b) for pre-post-pre (top left square), pre-pre-post (top right triangle) and post-pre-pre (left bottom triangle) combination of spikes produced by the first minimal TSTDP circuit. The circuit bias parameters for generating the synaptic weight changes shown in this figure correspond to the hippocampal bias set shown in Table 1.

As already mentioned, in 2002 Froemke and Dan proposed a suppression model for higher order spike trains and performed some experiments using the aforementioned extra triplet protocol. Their proposed suppression model can account for the required non-linearity in STDP experiments, when considering higher order of spike combinations. Fig. 5 shows that the first minimal TSTDP circuit, under the extra triplet protocol, and using the same set of parameters that were optimised for hippocampal experiments (shown in Table 1), is able to account for a similar behaviour to the experiments performed by Froemke and Dan in 2002 and for extra triplet patterns.

Although the proposed circuit implements the triplet model presented in [10] (and not the suppressive model in [13]), obtained results shown in Fig. 5 demonstrate qualitative regional agreement with the reported results in [13], nonetheless, there is a slight contrast between our results and their results in the post-pre-post case of spike patterns. Indeed, the triplet model weight changes induced by the pre-post-post, post-post-pre, pre-pre-post, and pre-post-post spike triplets are matched to the weight changes and result from the similar spike patterns obtained from the Froemke-Dan model. However, there is a slight difference in the results for pre-post-pre and a significant difference in the results for post-pre-post spike combinations when using these two different models. Right bottom square in Fig. 5(a), which represents the post-pre-post case, shows potentiation as it is the case for the post-pre-post spike pattern case in Fig. 4(b) also, however Froemke-Dan model results show a depression for this spike combination (Fig. 3b in [13]). According to the discussion provided in [10], the difference in the result is due to the nature of the original suppressive rule where post-pre-post contributions gave rise to a depression, in contrast to TSTDP where this specific combination leads to potentiation. Note that the Froemke-Dan revised model presented in 2006 addressed this issue, since in this model there are two different potentiation and depression saturation values [33]. This revised model now reproduces the expected experimental outcomes from [18].

#### Frequency-dependent pairing experiment

**Figure 6:**
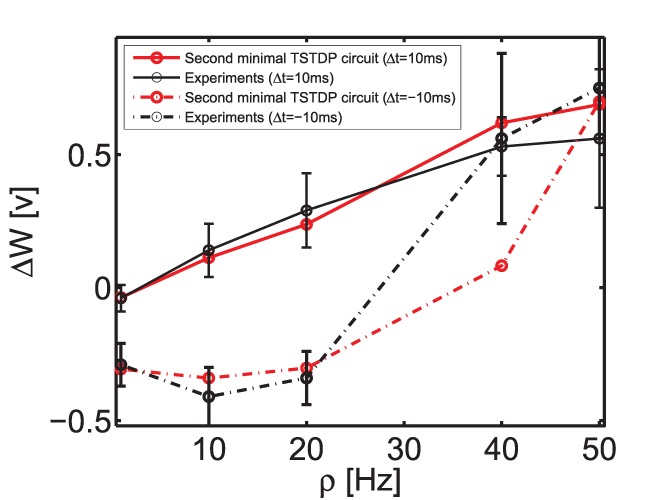
Frequency-dependent pairing experiment in the visual cortex region can be approximated using the minimal TSTDP circuit. Simulation results are produced under frequency-dependent pairing protocol and using the second minimal TSTDP circuit. The circuit bias parameters for generating the synaptic weight changes shown in this figure correspond to the visual cortex (second) set of bias parameters shown in Table 1. The experimental data shown in black are extracted from [10], [18].

As already mentioned, the frequency-dependent pairing experiments that were performed in the visual cortex, can also be replicated using a minimal TSTDP model. This model is simpler than the first minimal model and not only does not require the 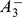 parameter from the full triplet model, but also it does not need the 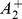 parameter (See Eq. 5). Hence, the minimal circuit for generating this experiment is also simpler from the first minimal circuit and does not include M7 (See Fig. 1). In order to approximate the outcome of frequency-dependent pairing experiments, which corresponds to the aforementioned visual cortex (second) data set, as reported in [10], [18], a new set of synaptic parameters for the model and therefore a new set of bias voltages for the circuit is required. As shown in Fig. 6, the optimised biases for the circuit can closely approximate the outcomes of experiments under frequency-dependent pairing protocol. The minimal obtained NMSE for this experiments was 0.39, which is close to the numerical simulation result of 0.34 reported in [10]. It is worth mentioning that the second minimal TSTDP circuit has only one transistor more than the simple PSTDP circuit proposed in [16], but it has the ability to reproduce the frequency-dependent pairing experiments, while all neuromorphic PSTDP circuits, even with much higher number of transistors (see [15], [22], [25] for example) fail to replicate these experiments [32].

#### BCM-like rate based experiment

**Figure 7:**
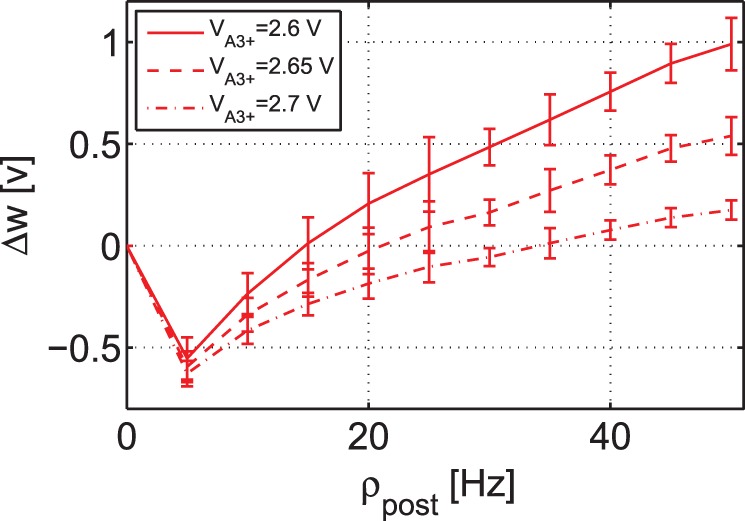
BCM-like behaviour with sliding threshold feature can be approximated using the minimal TSTDP circuit. Simulation results are produced under Poissonian protocol for BCM, and using the second minimal TSTDP circuit. The circuit bias parameters for generating the synaptic weight changes shown in this figure correspond to the visual cortex (second) set of bias parameters shown in Table 1. In this simulation, the pre-synaptic frequency, 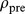 was kept fixed at 10 Hz, and the post-synaptic frequency, 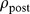 was swept (see the text for more details).

In addition to the outcome of frequency-dependent experiments, the second minimal TSTDP circuit is also able to account for a BCM-like behaviour. By employing the same circuit and set of bias parameters, which were used to generate frequency-dependent pairing experiments shown in Fig. 6, a BCM-like experiment is also reproducible. Fig. 7 depicts the synaptic weight changes produced by the second minimal TSTDP circuit and under the aforementioned Poissonian protocol. In this figure, three different curves show synaptic weight changes according to three different synaptic modification thresholds that demonstrate the points where LTD changes to LTP. The threshold is adjustable using the TSTDP rule parameters. In order to move the sliding threshold toward left or right, the 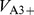 parameter can be altered as it is depicted in the figure. The rate of random pre-synaptic Poissonian spike trains, 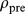, is equal to 10 Hz, and the trains with this spiking rate, are regenerated for each data point. Each data point shows the mean value of the weight changes for 10 various post-synaptic Poissonian spike trains and the error bars depict the standard deviations of the weight changes for each data points over 10 runs. In this experiment, similar to the experiment performed in [10], the frequency of the post-synaptic spike, 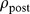 is swept over a range of frequencies from 0 Hz up to 50 Hz, while the pre-synaptic spiking frequency, 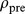 is kept fixed at 10 Hz.

**Figure 8:**
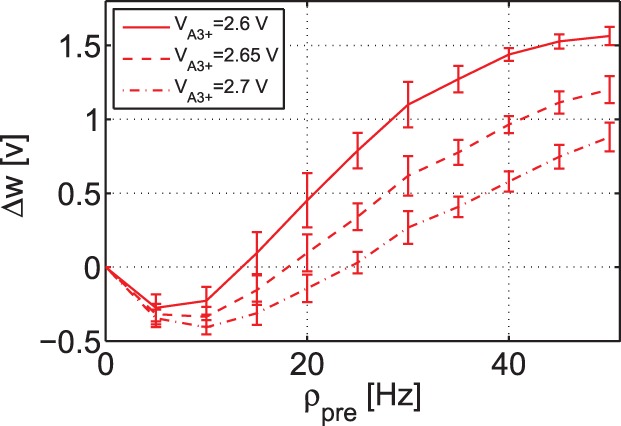
BCM-like behaviour with sliding threshold feature can be approximated using the minimal TSTDP circuit. Simulation results are produced under Poissonian protocol for BCM, and using second minimal TSTDP circuit. The circuit bias parameters for generating the synaptic weight changes shown in this figure correspond to the visual cortex (second) set of bias parameters shown in Table 1. In this simulation, the pre-synaptic frequency, 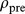 was swept, while the neuron is linear and 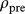 = 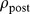 (see the text for more details).

Although Pfister and Gerstner have used this methodology to show that their model is able to reproduce a BCM-like behaviour, in the original BCM experiments reported in [34], the synaptic weight changes were measured whilst the pre-synaptic and not the post-synaptic spike rate was swept [9]. In order to check that the proposed circuit could reproduce BCM-like behaviour, which is driven by pre-synaptic activity, the circuit simulation was repeated. We made this simple assumption that post-synaptic firing rate is a linear function of the pre-synaptic firing rate, i.e. 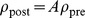 and for the sake of simplicity we let 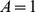, i.e 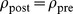. Despite such a crude approximation, the circuit is successfully able to mimic BCM-like behaviour where weight changes were pre-synaptically driven, as illustrated in Fig. 8. In this figure, each data point shows the mean value of the weight changes for 10 different trials using random Poissonian pre- and post-synaptic spike trains for each trial, and the error bars depict the standard deviations of the associated weight changes.

All these experiments suggest that the proposed timing-based circuit has a good ability to replicate the outcome of other synaptic plasticity experiments, for a BCM-like behaviour. In the next section we discuss and compare the proposed circuit and its counterparts from various circuit design as well as biological plausibility perspectives.

### Synaptic Plasticity Circuit Comparison

In order to measure the efficiency of the proposed analog neuromorphic circuit, it should be compared to its counterparts in terms of strength in reproducing the outcomes of various synaptic plasticity experiments. Besides, it is also essential to compare the proposed design with available synaptic plasticity circuits in various circuit design aspects such as required silicon real-estate, energy consumption, and process variation tolerance. In the following sections, we demonstrate that the proposed synaptic plasticity circuit outperforms most of its previous counterparts. In addition, it will be shown that the proposed circuit is much simpler, consumes less power and occupies smaller area in comparison to previous synaptic plasticity circuits. Furthermore, we show that the presented synaptic plasticity circuit is better than its counterparts in terms of process variation tolerance when a trade-off between complexity and performance is considered.

#### Synaptic plasticity ability for reproducing experimental data

As already mentioned, the proposed design is able to regenerate the outcomes of a variety of synaptic plasticity experiments. These experiments are not reproducible by any of the previous circuits designed for PSTDP rule. However, they can be replicated using a number of previously proposed TSTDP circuits, as well as a few other synaptic plasticity designs. Table 2 shows a detailed comparison among investigated circuits.

This table demonstrates that all PSTDP and TSTDP circuits are able to account for a BCM-like behaviour. However, simulation results presented in [35] suggest that, using a TSTDP circuit, a much nicer and smoother BCM-like behaviour is attainable and since there are more parameters available in the circuit, there will be a higher degree of control over the sliding threshold of the BCM rule. In addition, there is no evidence, if any of the circuits proposed in [36] or [24] are capable of showing a BCM-like behaviour with sliding threshold feature.

The table also summarizes the ability of the proposed TSTDP circuit in reproducing other required experiments. Although a number of other synaptic plasticity circuits that are shown in the table, are also capable of qualitatively generating the required experiments [23], [37], they need changes in their synaptic parameters or in their initial implementations, in order to be able to mimic biological experiments closely and with a small error. The table shows that the TSTDP designs proposed in [17], [32], [38] as well as the proposed design in this paper are able to account for all experiments using shared set of bias parameters. This is a useful feature of the synaptic plasticity circuit, to be able to reproduce as many experimental outcomes as possible, using a single set of parameters, and by means of a fixed design. As a result, this new plasticity circuit can be used in developing large-scale networks of spiking neurons with high synaptic plasticity abilities.

When implementing a large-scale network of spiking neurons, the synaptic plasticity circuits should be as area- and power-efficient as possible. This leads to the essential requirements of a large scale neuromorphic design, which include low power consumption and small area occupation. Despite these essential needs, most of the previously available synaptic plasticity VLSI designs do not meet these requirements. Some of these designs have good biological strength, but at the same time are large scale and power hungry such as the design presented in [17], [23], [24], [37], [38]. Some other designs such as the synaptic plasticity circuits presented in [15], [16], [22], [25], [39], have improved power and area features, but do not have most of the required biological abilities. Therefore, a circuit with low power and area consumption and at the same time with high synaptic plasticity capabilities is required. The design presented in this paper aims at reaching these goals. This design has high synaptic weight modification ability, while it is low power and occupies small silicon area.

#### Area and power consumption

Since the proposed design only uses a small number of transistors to reach its required synaptic plasticity features compared to many previous designs with less or equal synaptic capabilities, the area and power consumption in this design are lower than all previous designs with similar capabilities, and close to other design with much less synaptic strength. Table 3 compares the proposed design, with some of the previous synaptic plasticity designs available in the literature, in terms of complexity (required number of transistors and capacitors), which has a direct relation with the needed silicon area, and their estimated power consumption.

**Table 3:** Area and power comparison for various synaptic plasticity circuits.

Plasticity Circuit\Comparison Measure	Transistor No.	Capacitor No.	Energy per spike	NMSE*
PSTDP [25] with weight dependence	15	5	0.3 pJ	>10
PSTDP [39] **	>100	4	0.37 pJ	>10
PSTDP [22] ***	18	3	42 pJ	>10
Voltage-based BCM [23]	>100	2	NA	NA
Iono-neuromorphic [24]	>100	2	NA	NA
Iono-neuromorphic [37]	>100	2	NA	NA
PSTDP [15] without weight dependence part	15	3	1.5 pJ	10.76
PSTDP [16]	12	1	3 pJ	11.3
TSTDP [32]	26	1	0.03 pJ	3.46
TSTDP [38]	44	7	1.5 pJ	2.25
TSTDP [17]	37	5	60 pJ	1.74
Proposed minimal TSTDP	14	1	0.02 pJ	2.04

*The biases are optimised for the hippocampal (first) data set and then the energy consumptions are measured.

**This design has been implemented in a 90 nm CMOS process with a supply voltage of 0.6 V.

***This design has been implemented in a 0.25 µm CMOS process, while power supply = 3.3 V has been equal to the other presented designs in this table.

Power consumption of a synaptic plasticity circuit is directly linked to its synaptic biasing parameters such as its synaptic time constants e.g. 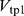, 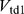, 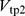, 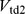, as well as its synaptic amplitude parameters e.g. 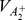, 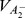, 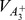, 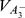. In addition, consumed power is in a direct relation with the supply power, as well as the spike pulse width. Therefore, in order to have a fair comparison among synaptic plasticity circuits, they should all be compared under similar conditions. The presented results in the last six rows of Table 3, depict the simulation results for various circuits under similar conditions. The synaptic parameters, for all these synaptic circuits are firstly optimised to reach the best NMSEs for the hippocampal data set. The optimisation process determines the value of synaptic biasing parameters, which significantly influence the power consumption of these circuits. For instance, the high power consumption observed in the TSTDP circuit proposed in [17] is due to large time constants required for reaching a small 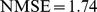, which results in transistors being on for longer period of time and this leads to high power consumption. Table 3 reports the energy consumption per spike for a number of the mentioned designs. The energy consumption is measured on both pre-synaptic and post-synaptic spikes. Due to differences in depressions and potentiations biasing parameters, different energy consumptions are measured for pre- and post-synaptic spikes, but the larger one is reported in Table 3.

The energy consumption per spike for the first three designs in Table 3, are extracted from related papers. These circuits are PSTDP circuits, which do not posses the high biological plausibility available in TSTDP circuits including the low power TSTDP design presented in this paper. Although two of these designs are low power and consumes very low energy per spike, they require a high number of transistors/capacitors that require large silicon area. Note that in the best case, the NMSE of these designs that implement the same STDP rule as the design presented and simulated in [32], [38], will be >10, which is not acceptable as a fitting error.

In addition, there is no energy consumption information available for the other three designs shown in the fourth to sixth rows of the table. Two of these designs are biophysically-based synaptic plasticity circuits, which are bulky detailed VLSI circuits implemented with more than 100 transistors, and the other one that implements the voltage-based BCM rule, imposes an inevitable interference with the neuron circuit and also needs more than 100 transistors for the design [5], [23].

Considering both area and power consumption, under similar conditions to other synaptic plasticity circuits, Table 3 suggests that the proposed design outweighs all other designs in terms of energy consumption, silicon real estate, and biological accuracy.

In addition to operating the transistors in the subthreshold region of operation, which makes the proposed circuit low-power, the accelerated time scale is another factor that results in a lower energy consumption, compared to other designs, which are implemented on real time scales. This is due to the fact that the static current, which is usually the dominant power consumption cause, is reduced [8].

This allows the proposed design to be a suitable learning and computational component for large scale and low power neuromorphic circuits with high biological capability. However, one should keep in mind that, any analog VLSI design will be affected by the mismatch due to fabrication imperfections. Therefore, besides area and energy consumption, mismatch may also be taken into account when considering design of an analog synaptic plasticity circuit for learning and computational purposes.

#### Process variation and transistor mismatch

Apart from power consumption and silicon area, transistor mismatch is another challenge that is always associated with all analog VLSI designs, specially designs for synaptic plasticity circuits. The functionality of these circuits are dependent on the synaptic parameters and changes in the values of these parameters, which can happen due to process variations, results in deviation from the synaptic circuit expected behaviour. These deviations can bring about degradation of synaptic plasticity capability. The mismatch may be taken into account from two different design perspectives. First, is a mismatch that occurs between the targeted design and the implemented design, and results in the physically implemented transistor to be different from the designed one. Second, is a mismatch that occurs among the transistors all over the fabricated design. These transistors suppose to have similar behaviour and functionality inter- or intra-chip. The design of large neuromorphic circuits become challenging due to these mismatches.

Transistor mismatch becomes more challenging when the transistor works in its subthreshold region of operation. This is due to the changes to the threshold of the transistor, and therefore affect its subthreshold current characteristics. Due to the exponential behaviour and also low power consumption of transistors in their subthreshold regime, many spiking neural circuits, including neurons and synaptic weight change components are implemented in this region. In addition, many neuromorphic VLSI designs employ mismatch susceptible components such as current mirrors and differential pairs in their current- or voltage-mode structures. Therefore, these neural systems are seriously susceptible to device mismatches [17], [23], [38], [40].

There are various approaches to reduce the transistor mismatch problem in Neuromorphic VLSI design. These approaches include (i) fine-tuning the design after fabrication [17], [41], (ii) alleviating the device mismatch [21], [42], (iii) exploiting the device mismatch for neural learning [43], (iv) utilising newly developed threshold voltage variation tolerant processes [44] for ultra-low-power subthreshold neuromorphic designs [24], [40], and (v) wide dynamic range neuromorphic circuit design approach that employs source degeneration and other negative feedback design techniques to increase the dynamic range of the input voltages to the neuromorphic circuits and therefore decrease their vulnerability to device mismatches [37], [40], [45].

Each of these approaches has its own advantages and disadvantages. For instance the approach used in [24], [37] requires specially designed process tolerant circuits with negative feedbacks and source degeneration features, which lead to increased number of transistors and therefore result in larger circuits. In addition, the fine-tuning approach that has been successfully utilised in [17], is not applicable for large-scale neuromorphic circuits. Nonetheless, this approach could be used for a set of circuits with shared synaptic parameters across the chip, or even inter-chips, in order to reach the required functionality.

**Figure 9:**
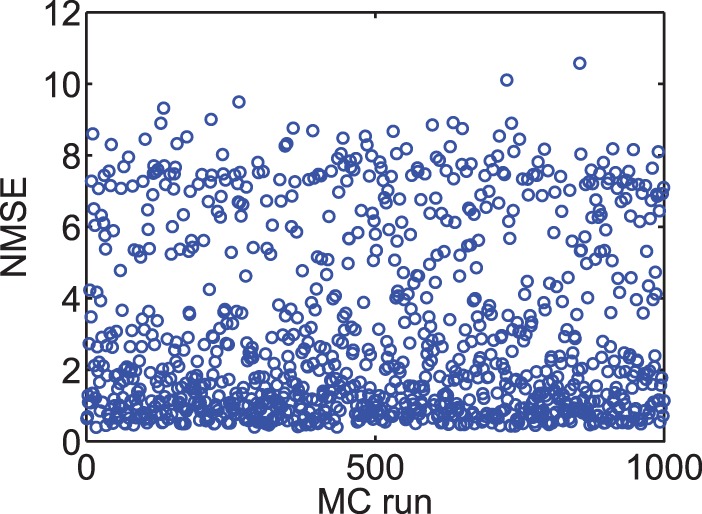
Transistor mismatch effects on the proposed design. This figure shows 1000 Monte Carlo (MC) runs. In each run, the threshold voltage of all transistors are independently varied, based on a three-sigma deviation. The NMSE in each MC run shows the fitting error of the design, which is affected by that run deviated transistors thresholds. Similar to Fig. 6, simulation results are produced under frequency-dependent pairing protocol and using the second minimal TSTDP circuit. The circuit bias parameters correspond to those for the visual cortex region shown in Table 1.

In order to have a process tolerant design, it is essential to use less components susceptible to mismatch including current mirrors [17], [38], differential pairs [46], and OTAs [39], [47]. The proposed design in this paper does not use any of these components and it is less susceptible to process variations than many previous designs. Fig. 9 shows the variation in NMSE for visual cortex data set, when a rigorous case mismatch scenario happens in the fabrication. In the applied scenario, all transistors in the design independently go under a 1000 Monte Carlo (MC) threshold voltage variation, with three standard deviations from their typical process technology threshold voltage. This may cause deviations in the threshold voltage of any transistors up to 30 mv. This level of variation in the thresholds of transistors is very unlikely to occur. This variation scenario was used in a previous design proposed in [17], where under the same protocol the worst case NMSE can go up to 80 (See Fig. 13 in [17]). So the proposed design is much more stable compared to the previous designs and that is because of not using of process variation susceptible circuit modules, such as current mirrors, which are extensively used in the previous designs (See Fig. 1 of [17], as well as Fig. 2 of [38]). Note that the circuit bias parameters for all 1000 MC runs are fixed and correspond to the parameters for visual cortex parameters shown in Table 1. However, as the results presented in [17] show, the bias parameters can be justified again and bring the circuit back to a significantly low NMSE.

**Figure 10:**
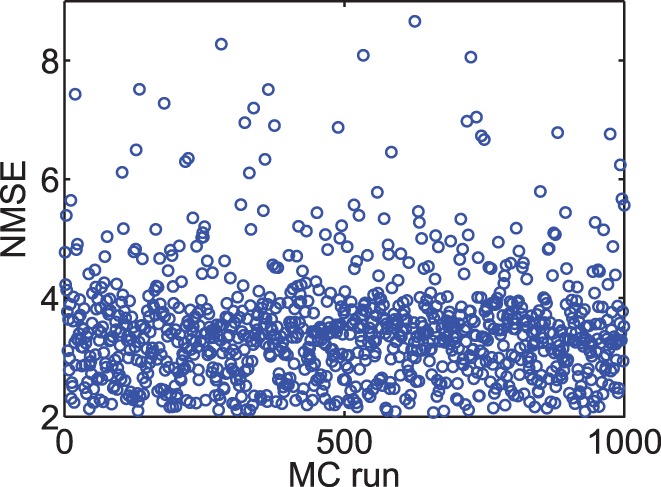
Transistor mismatch effects on the proposed design. This figure shows 1000 Monte Carlo (MC) runs. In each run, the threshold voltage of all transistors are independently varied, based on a three-sigma deviation. The NMSE in each MC run shows the fitting error of the design, which is affected by transistor threshold deviation. Simulation results are produced under pairing, triplet and quadruplet protocols and using the first minimal TSTDP circuit. The circuit bias parameters correspond to those for the hippocampal region shown in Table 1.

Identical to the mismatch analysis performed in Fig. 9, the proposed TSTDP circuit is subjected to another variation analysis, this time using the first minimal TSTDP circuit and while stimulated under the pairing, triplet and quadruplet experiments, in order to measure the variation effect. Fig. 10 represents 1000 MC runs, and the NMSE deviation, for the mismatch scenario explained earlier. The NMSE obtained using the new proposed circuit is significantly smaller than that of the design presented in [17], [38].

According to Figures 9 and 10, in both cases of mismatch analysis, more than 60% of NMSEs are very close to the best reached NMSEs in simulations. In addition, even the worst NMSEs shown in these figures that are due to severe unlikely mismatch, are still better than PSTDP circuit NMSEs even without considering variation in them. Furthermore, it should be noted that, the applied variation scenario considers independent changes in the design. This means that the threshold voltage of every single transistor in the design changes independently, which is not likely in the case of closely positioned transistors in the proposed compact design. Considering this fact a mismatch tolerant synaptic circuit design is expected after fabrication. However, these independent changes can happen globally and in the replicates of the proposed plasticity circuit across the chip, in the case of a large scale neuromorphic design. This means that shared fine-tuning for various sets of synaptic circuits, which are positioned in a close neighbourhood on the chip, could be an effective way of tackling the mismatch problem.

In general, Figures 9 and 10 suggest that the proposed circuit is not heavily affected by process variation, and an acceptable synaptic behaviour compatible with several synaptic plasticity protocols is expected after fabrication. This feature along with low power consumption, small area requirement, and high biological accuracy, make the proposed circuit an ideal synaptic plasticity component that can be utilised in large scale neuromorphic systems. These systems will have higher capability to mimic more biological experiments, while enjoying a compact structure, which consumes little power. This is significant progress toward developing biologically plausible systems on scales approaching that of the brain.

## Limitations of Study and Future Works

Despite the performance advantages that the proposed circuit presents, it has a number of limitations that need to be considered when integrating it within a network configuration. As Fig. 1 demonstrates, in order to induce weight changes using the triplet circuit, current pre- or post-synaptic spike i.e. 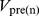 or 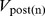, as well as the immediate previous pre- or post-synaptic spike i.e. 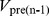 or 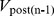 are needed. This results in the need for introducing a delay into the design that provides the circuit with a delayed version of pre- and post-synaptic spike trains.

In our simulation setup, we have delayed the input pre- and post-synaptic spike trains, generated in software, for one spike width of 1 µs, and produced the required delayed spike trains, i.e. 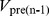 and 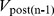. However, in the physical implementation of the proposed TSTDP circuit, the mentioned delay element should be combined with either neuron or synapse circuit, in order to produce the required delayed spike trains. Since the density of neurons is significantly lower than that of synapses in a neuromorphic system, it is therefore preferred to integrate the required delay element into the neuron design, hence saving precious silicon real estate and reduced power consumption. Another viable method for implementing a delay into the system is to delay the spike while transmitting it via an Address Event Representation (AER) protocol in the system. Since in the AER, we only transfer spike time stamps, we can easily delay the spike time for any specified value. Because the AER is an unavoidable part of any neuromorphic system, it is beneficial to use AER instead of any extra circuitry (whether part of the neuron or synapse) for introducing the required delay times into the system.

Another limitation in the proposed circuit is the use of a large weight capacitor, in order to retain the synaptic weight for required period of times, needed for adopted experimental protocols. The utilised capacitor can be implemented using Metal Oxide Semiconductor Capacitors (MOSCAPs), which approximately consumes up 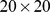 µm^2^ of silicon real estate. Therefore, compared to the Full TSTDP circuit body that is composed of 18 transistors all with 1.05 µm width and 0.35 µm length, the capacitor takes up about 90% of the whole area required for the TSTDP circuit.

In a recent study we have shown that a similar version of the proposed circuit can use a 50 fF capacitor instead of the very large 1 pF one, while retaining its ability to reproduce the STDP learning window, and the triplet and quadruplet experimental data [48]. This becomes possible if we use a modified version of the experimental protocols, which consider only one pair, triplet or quadruplet of spikes, instead of the original protocols that use 60 spike sets with a frequency of 1 Hz. The design in [48], cannot account for the frequency-dependent pairing experiments, or other complicated experiments shown in this paper, and is suitable only for experiments with high spike frequencies. On the contrary, the utilised experimental protocols in this paper introduce 60 pairs, triplet, or quadruplet of spikes with frequency of 1 Hz, into the TSTDP circuit, and the resulting weight change is the summation of the weight changes of all these 60 spike sets. Therefore, the synaptic weight change after each of these spike sets should be strongly preserved during the rest period before the arrival of the next spike set, or for longer times when there is no spike. However, due to the capacitor leakage, the synaptic weight stored on the capacitor, will leak away resulting in the learnt weight will be eventually altered/lost. This is the reason why we have used a large capacitor in our design to minimise this loss. Similarly, many of the previous designs [15], [16], [25], which only possess synaptic weight changes for the STDP protocol, with only one spike pair, also utilised large capacitors, for the same reason.

However, with large capacitors, and even accelerated time, the leakage current still has a significant effect on the stored synaptic weight value. In the performed simulations throughout this paper, we have reported the voltage difference between the synaptic weight values stored on the capacitor, at the start of the experiments and just after the experiment is finished. During the experiment, the leakage is not significant and can be compensated for, using the parameter tuning performed for the TSTDP circuit. However, after the experiment is finished, namely when there is no spike coming, the updated weight stored on the capacitor will leak away in less than a second. For an example, see the STDP measurement results from a similar accelerated-time neuromorphic chip reported in [8].

In order to save the latest weight status of the synapse after learning, its weight can be categorized into two potentiated/depressed states, if the weight on the capacitor is above/below a predetermined threshold. This is a bistability mechanism such as the one utilised in [16] and can be employed along with our circuit, so that the synaptic weight will be either potentiated or depressed, depending on the latest changes TSTDP circuit made on the synaptic weight. In this condition, since the synaptic weight is quantised into a binary high (potentiated) or low (depressed) state, it loses its analog value. Although this approach results in a decrease in the synaptic weight capacitor size, it compromises the analog nature of the synaptic weight, which may be essential for some specific applications, where high degree of synaptic weight precision is necessary. In future work we suggest the use of TSTDP synapses that are driven to two bistable states, using a bistability circuit similar to the one used in [16]. This may lead to further reduction in the size of the weight capacitor, hence, the area of the TSTDP synapse.

Note that, even with the use of a bistable mechanism, the final synaptic weight ought to be in a nonvolatile storage element for later use. Therefore, there is always need for long-term synaptic weight storage. There exist a number of nonvolatile weight storage methods in neuromorphic engineering such as (i) memory cells [41], (ii) floating gate [49], and (iii) memristive devices [50], which could be utilised for this task.

## Conclusion

A low-power, compact, and tunable neuromorphic circuit with high synaptic plasticity capabilities is proposed. Simulation results demonstrate how the proposed circuit can mimic the outcomes of several biological synaptic plasticity experiments. The presented design is compared with many previous synaptic plasticity circuits, in terms of power consumption, area consumed, biological accuracy, and tolerance to transistor mismatch and process variation. The comparison of results shows that the proposed circuit possesses good synaptic plasticity capabilities that can be used in the implementation of large scale neuromorphic systems, which may potentially lead to neuromorphic systems with higher learning and computational abilities.
